# Intravenous iron therapy for heart failure and iron deficiency: An updated meta‐analysis of randomized clinical trials

**DOI:** 10.1002/ehf2.14905

**Published:** 2024-07-04

**Authors:** Mushood Ahmed, Aimen Shafiq, Hira Javaid, Priyansha Singh, Haania Shahbaz, Muhammad Talha Maniya, Hritvik Jain, Najwa Shakir, Huzaifa Ahmad Cheema, Adeel Ahmad, Wajeeh Ur Rehman, Gabriel Yeap, Abdulqadir J. Nashwan, Abdul Mannan Khan Minhas, Raheel Ahmed, Marat Fudim, Gregg C. Fonarow

**Affiliations:** ^1^ Department of Medicine Rawalpindi Medical University Rawalpindi Pakistan; ^2^ Department of Medicine Dow University of Health Sciences Karachi Pakistan; ^3^ Department of Medicine Allama Iqbal Medical College Lahore Pakistan; ^4^ Smt. Nathiba Hargovandas Lakhmichand Municipal Medical College Ahmedabad India; ^5^ Department of Medicine Ziauddin University Karachi Pakistan; ^6^ All India Institute of Medical Sciences (AIIMS) Jodhpur India; ^7^ Department of Cardiology King Edward Medical University Lahore Pakistan; ^8^ Department of Internal Medicine Mass General Brigham ‐ Salem Hospital Salem Massachusetts USA; ^9^ Department of Internal Medicine, Binghamton Clinical Campus SUNY Upstate Medical University Binghamton New York USA; ^10^ Faculty of Medical Sciences Newcastle University School of Medicine Newcastle upon Tyne UK; ^11^ Hamad Medical Corporation Doha Qatar; ^12^ Section of Cardiovascular Research Baylor College of Medicine Houston Texas USA; ^13^ National Heart & Lung Institute Imperial College London London UK; ^14^ Department of Cardiology Royal Brompton Hospital London UK; ^15^ Department of Medicine Duke University Medical Center Durham North Carolina USA; ^16^ Duke Clinical Research Institute Durham North Carolina USA; ^17^ Ahmanson‐UCLA Cardiomyopathy Center, Division of Cardiology University of California Los Angeles Los Angeles California USA

**Keywords:** Ferric carboxymaltose, Heart failure, Iron deficiency, Intravenous iron

## Abstract

Heart failure (HF) patients frequently exhibit iron deficiency, which is associated with a poor prognosis. Although various trials have been conducted, it is uncertain if intravenous (IV) iron replenishment improves clinical outcomes in HF patients with iron deficiency. A comprehensive literature search was conducted using PubMed/MEDLINE, Embase, and the Cochrane Library from inception till 15 September 2023 to retrieve randomized controlled trials (RCTs) that compared IV iron therapy with placebo or standard of care in patients with HF and iron deficiency. Clinical outcomes were assessed by generating forest plots using the random‐effects model and pooling odds ratios (ORs) or weighted mean differences (WMDs). Fourteen RCTs with 6651 patients were included. IV iron therapy showed a significantly reduced incidence of the composite of first heart failure hospitalization (HHF) or cardiovascular (CV) mortality as compared with the control group (OR = 0.73, 95% CI: 0.58 to 0.92). The IV iron therapy resulted in a trend towards lower CV mortality (OR = 0.88, 95% CI: 0.76 to 1.01), 1‐year all‐cause mortality (OR = 0.85, 95% CI: 0.71 to 1.02), and first HHF (OR = 0.73, 95% CI: 0.51 to 1.05), and an improved left ventricular ejection fraction (LVEF) (MD = 4.54, 95% CI: −0.13 to 9.21). Meta‐regression showed a significant inverse moderating effect of baseline LVEF on the first HHF or CV death. In patients with HF and iron deficiency, IV iron therapy reduced the incidence of composite of first HHF or CV mortality. There was a trend of lower overall CV and 1‐year all‐cause mortality, first HHF, and improved LVEF with IV iron therapy.

## Introduction

Nearly 50% of individuals with heart failure (HF) have concomitant iron deficiency.^1^, ^2^ An insufficient supply of iron affects cellular energetics and oxidative metabolism, which has a detrimental impact on the heart and skeletal muscles.^3^, ^4^ Irrespective of the presence of concurrent anaemia, this culminates in substantially decreased exercise capacity and a poor quality of life (QoL) and is linked to an elevated risk of mortality.^5^, ^6^ Considering these factors, the present European Society of Cardiology (ESC) guidelines advocate routine testing of HF patients for iron deficiency.^7^ Additionally, ESC and American Heart Association/American College of Cardiology (AHA/ACC) guidelines endorse intravenous (IV) iron therapy in HF patients to facilitate the improvement of symptoms, exercise endurance, and QoL.^7^, ^8^

Past studies have consistently identified that patients receiving IV iron therapy had enhanced exercise endurance and QoL compared with a placebo.^9^, ^10^ Additionally, numerous trials have been conducted to determine the impact of IV iron on hospitalizations and mortality; however, they have produced inconsistent results. Contrary to the FAIR‐HF study,^9^ which was potentially overly brief in duration, the CONFIRM‐HF trial^10^ discovered a statistically significant reduction in overall HF hospitalizations with IV iron. The AFFIRM‐AHF study^11^ evaluated IV iron in HF patients and observed a significant decrease in overall HF hospitalizations. Still, the reduction in total HF hospitalizations and cardiovascular (CV) death remained insignificant, with point estimates favouring IV iron therapy.^11^

Hence, it is still undetermined if IV iron replenishment reduces CV mortality and HF hospitalizations. Although previous meta‐analyses^12^, ^13^ have been conducted on this topic, which showed a reduction in HHF, the results were inconclusive regarding CV or all‐cause mortality. Hence, to address this literature gap, we conducted this meta‐analysis to pool all the studies published to date, including the HEART‐FID trial,^14^ which is the most recent and the largest clinical trial on this topic, to provide the most comprehensive and robust evidence regarding the clinical outcomes of IV iron therapy in HF patients.

## Methods

This systematic review and meta‐analysis observed the guidelines established by the Preferred Reporting Items for Systematic Review and Meta‐Analysis (PRISMA).^15^ The study was registered with PROSPERO: CRD42023471054.

### Data sources and search strategy

Two authors (M. A. and H. J.) independently searched PubMed/MEDLINE, Embase, the Cochrane Library, and ClinicalTrials.gov from their inception until 15 September 2023, with no language restrictions. Additionally, the reviewers manually examined references from retrieved trials, previous meta‐analyses, and review articles to ensure that all relevant studies were included. The search string used consisted of the following keywords and related Medical Subject Headings (MeSH) terms: (((heart failure) OR (systolic heart failure) OR (diastolic heart failure)) AND ((iron deficiency)) OR (iron repletion)) OR (intravenous iron)) OR (iron sucrose)) OR (ferric derisomaltose)) OR (ferric carboxymaltose)) OR (iron isomaltoside1000)) OR (sodium ferric gluconate complex)) OR (iron supplementation)) OR (iron therapy))).

### Eligibility criteria and outcomes

The studies were eligible for our systematic review and meta‐analysis if they: (i) were randomized controlled trials (RCTs) with a follow‐up duration of at least 2 weeks; (ii) included HF patients with iron deficiency; (iii) had adult male or female participants who were at least 18 years old; (iv) compared IV iron with either standard care or placebo; (v) evaluated at least one of the predetermined efficacy and safety outcomes. We excluded trials that used oral iron therapy. Other studies excluded from our analysis included those with insufficient data, case reports, case series, letters and editorials, and reviews.

The primary outcomes were the composite outcome of first hospitalization for HF (HHF) and CV death, overall all‐cause mortality, and overall CV mortality. The secondary outcomes were overall mortality and CV mortality at 1 year, first HHF, change in left ventricular ejection fraction (LVEF), improvement in New York Heart Association (NHYA) class from baseline, distance covered in meters in 6‐min walk test, and adverse events. The definitions of all pooled outcomes are provided in Table S1.

### Study selection and data extraction

All the studies from the literature search were imported to EndNote X9 (Clarivate Analytics), and duplicates were identified and removed. Two authors (M. A. and H. J.) independently reviewed trials based on their titles and abstracts. The full texts of the articles were then examined, and any study that conformed to our eligibility criteria was included. In cases of disagreement, a third author (P. S.) was consulted.

The following data were extracted for each study: author surname, year, mean patient age, main inclusion criteria, CV risk factors (hypertension, diabetes, and atrial fibrillation), iron profile (mean haemoglobin levels, mean ferritin, and mean saturation), formulations of IV iron, and mean duration of follow‐up. A pre‐piloted Excel sheet was used for data extraction.

### Quality assessment of included studies

Version 2 of the Cochrane Risk of Bias tool for RCTs was used to assess the risk of bias.^16^ Risk of bias was assessed across five domains: randomization, deviations from intended variation, missing outcome data, measurement of outcome, and selection of reported results. The trials were scored as high, with some concerns, or low risk of bias in each domain.

### Statistical analysis

Data analysis was performed using RevMan, Version 5.4 (Nordic Cochrane Center, Copenhagen, Denmark) and Comprehensive Meta‐Analysis Version 3.0. The results were presented as odds ratio (OR) and mean difference (MD) for the dichotomous and continuous outcomes, respectively, with 95% confidence intervals (CIs). The results were pooled using the DerSimonian and Laird random‐effects model^17^ and visualized using forest plots. Heterogeneity across the trials was assessed using the Higgins *I* ^2^ test; a value <25% indicates low heterogeneity, 25–75% indicates moderate heterogeneity, and >75% indicates high heterogeneity.^18^ For the primary outcomes, we conducted a subgroup analysis based on the IV iron formulations (ferric carboxymaltose vs. other IV iron forms). A meta‐regression analysis was performed for the primary outcomes to determine the effect of the baseline LVEF and follow‐up duration (weeks) on the pooled estimates calculated using a random‐effects model in our analysis. Meta‐regression scatter plots were generated to evaluate the meta‐regression analysis results visually. The publication bias was assessed through a visual inspection of funnel plots and Begg's rank test. A *P*‐value of <0.05 was considered significant in all cases.

## Results

**Figure 1:**
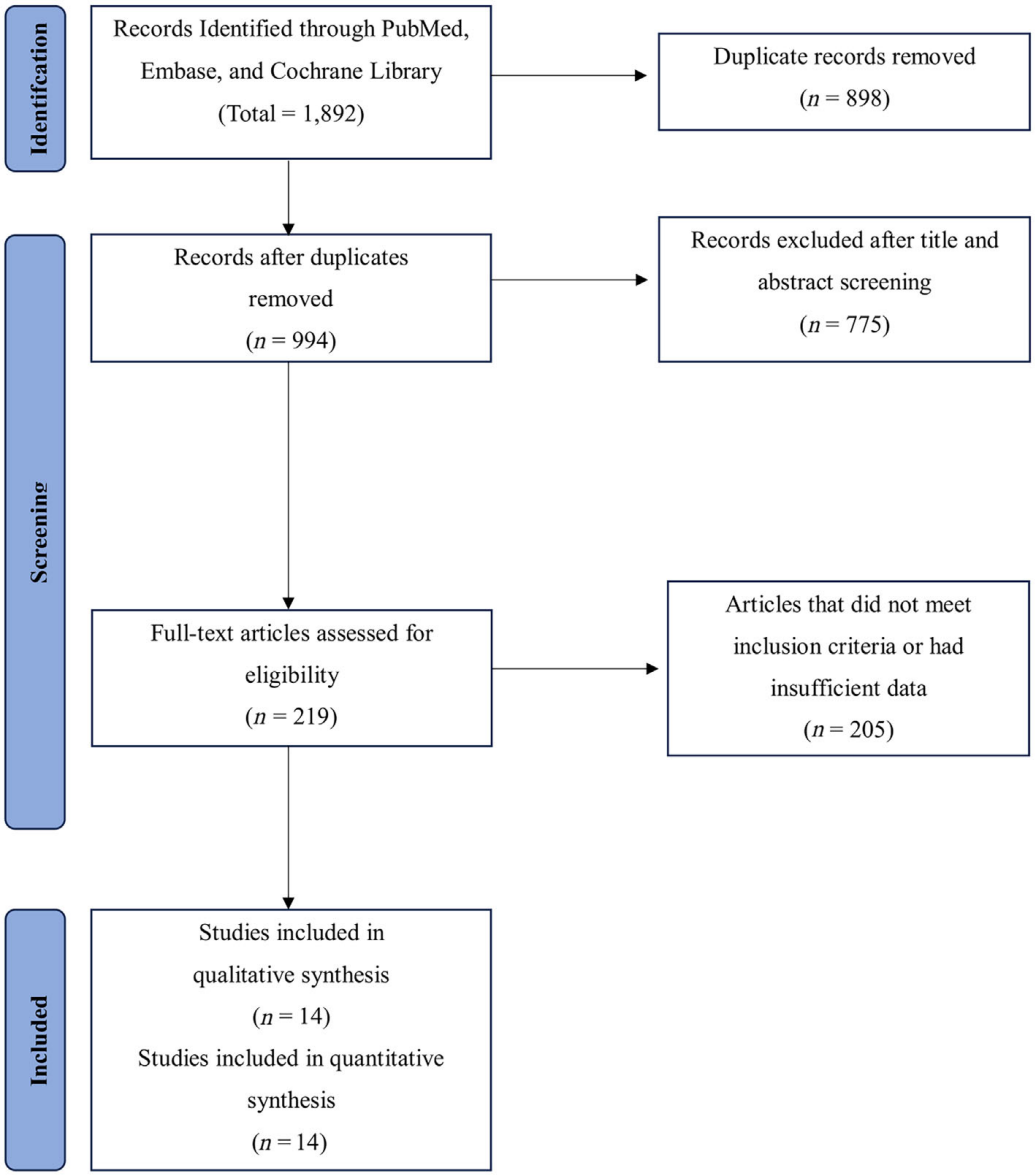
PRISMA flowchart of included studies.

The systematic literature search yielded 1892 records. After screening, 14 RCTs were eligible to be included in our meta‐analysis. The PRISMA flowchart depicts the study selection and screening process, as shown in *Figure* *1*.

### Study characteristics

Fourteen RCTs^9^, ^10^, ^11^, ^14^, ^19^, ^20^, ^21^, ^22^, ^23^, ^24^, ^25^, ^26^, ^27^, ^28^ were pooled in our study, with a total of 6651 participants. The publication years of the included trials ranged from 2007 to 2023. The number of patients in the treatment group who received IV iron was 3412, and the number of patients in the control group was 3239. The studies varied in sample sizes, type of IV iron administered, baseline LVEF%, and follow‐up durations. The participants' mean age ranged from 51 to 75.4 years, with males making up the majority of the study population in 12 studies. The mean follow‐up duration was 33.85 weeks. Eight studies used ferric carboxymaltose^9^, ^10^, ^11^, ^14^, ^25^, ^26^, ^27^, ^28^; three studies used iron sucrose^20^, ^21^, ^24^; and ferric derisomaltose,^19^ sodium ferrate gluconate complex,^22^ and iron isomaltoside^23^ were administered in one study. The detailed characteristics of the included studies and patients are given in *Table* *1*.

**Table 1:** Characteristics of included studies

Author/trial name	Year	IV iron (*n*)	Placebo/SOC (*n*)	Mean age (SD/IQR)	Females (%)	Baseline LVEF (%)	Main inclusion criteria (mg/dL; %)	Cardiovascular risk factors			Iron profile			Form of iron	Follow‐up duration weeks (IQR)
								HTN (%)	DM (%)	Afib (%)	Mean Hb; g/dL (SD/IQR)	Mean ferritin (SD/IQR/median)	Mean TSAT % (SD/IQR/median)		
Marcusohn E et al.	2022	19	15	IV 70 (64.0–76.2) C 74.5 (70.5–82.5)	IV 33.3 C 31.2	IV = 30% (IQR, 15–50) C = 40% (IQR, 28–58)	HHF Hb 8–14	IV 94.4 C 93.8	IV 61.1 C 75.0	IV 55.6 C 31.3	IV 11.9 (10.8–12.5) C 11.4 (110.2–12.5)	IV 92 (50–165) C 110 (55–177)	IV 12.6 (9.5–14.8) C 14.0 (10.1–16.7)	SFGC	24
FERRIC‐HF II (Edwards et al.)	2019	21	19	IV 70 ± 12 C 62 ± 13	IV 24 C 32	IV = 37 ± 8 C = 37 ± 8	NYHA II/III LVEF ≤ 45 Hb < 13	IV 62 C 64	IV 48 C 53	IV 29C 21	IV 130 ± 15 C 128 ± 20	IV 34 (18–50) C 59 (39–79)	IV 21 ± 8 C 18 ± 10	IIM	2
Toblli et al. 2015	2015	30	30	IV 75.4 ± 6 C 74.7 ± 7	IV 53.3 C 56.6	IV = 30.2 ± 3.5 C = 29.9 ± 3.2	LVEF ≤ 35 NYHA II/III/IV Hb < 12.5	NA	NA	NA	IV 10.1 ± 0.8 C 10.1 ± 0.6	IV 70.6 ± 24.9 C 68.4 ± 18.3	IV 19.2 ± 1.8 C 19.2 ± 1.9	IS	26
HEART FID (Mentz et al.)	2023	1532	1533	IV 68.6 ± 10.9 C 68.6 ± 11.2	IV 33 C 34.6	IV = 30.8 ± 7.0 C = 30.6 ± 7.3	LVEF ≤ 40 Hb > 9–15	NA	IV 45.3 C 45.1	IV 44.1 C 43.3	IV 12.6 ± 1.4 C 12.5 ± 1.4	IV 56.0 ± 47.3 C 57.3 ± 51.4	IV 23.9 ± 11.2 C 23.0 ± 10.3	FCM	52
Toblli et al 2007	2007	20	20	IV 76 ± 7 C 74 ± 8	NA	IV = 31.3 ± 3.7 C = 30.8 ± 1.7	LVEF ≤ 35 NYHA II/III/IV Hb ≤ 12.5	NA	NA	NA	IV 10.3 ± 0.6 C 10.2 ± 0.5	IV 73.0 ± 29.9 C 70.6 ± 21.4	IV 20 ± 1 C 20 ± 1	IS	24
FERRIC HF (Oknoko el al.)	2008	24	11	IV 64 ± 14 C 62 ± 11	IV 29 C 27	IV = 30 ± 7 C = 29 ± 6	NYHA II/III Hb ≤ 12.5 LVEF ≤ 45	IV 50 C 45	IV 33 C 36	NA	IV 12.6 ± 1.2 C 12.2 ± 1	IV 62 ± 37 C 88 ± 62	IV 20 ± 8 C 21 ± 9	IS	18
FCM‐HF‐IN (Dhoot et al.)	2020	35	35	IV 51.0 ± 11.6 C 54.8 ± 9.0	IV 42.9 C 40	IV = 26.6 ± 4.9 C = 27.1 ± 5.4	NYHA II/III Hb > 8	NA	NA	NA	IV 11 ± 1.4 C 11.3 ± 0.9	IV 40.1 ± 27.2 C 45.5 ± 35.1	NA	FCM	12
PRACTICE‐ASIA‐HF (Yeo el al.)	2018	25	25	IV 61.1 ± 10.8 C 64 ± 10	IV 25 C 20	IV = 38.8 ± 17.5 C = 33.2 ± 14.8	HHF Hb ≤ 14	IV 87.5 C 72	IV 62.5 C 60	NA	IV 11.6 ± 1.9 C 13.1 ± 1.3	IV 91.4 ± 80.4 C 84.1 ± 63.7	IV 15.7 ± 10.1 C 13.9 ± 6.8	FCM	12
EFFECT‐HF (Veldhuisen et al.)	2017	86	86	IV 63 ± 12 C 64 ± 11	IV 30 C 20	IV = 33 ± 9 C = 31 ± 8	LVEF ≤ 45 NYHA II/III Hb < 15	IV 72 C 65	IV 30 C 37	IV 41 C 48	IV 12.9 ± 1.3 C 13.0 ± 1.5	IV 48 C 53	IV 17.3 C 18.1	FCM	24
IRON‐CRT (Martens et al.)	2021	37	38	IV 72 ± 12 C 73 ± 9	IV 30 C 34	IV = 33 ± 8 C = 34 ± 7	CRT > 6 months LVEF ≤ 45 NYHA II/III/IV	IV 87 C 97	IV 46 C 50	NA	IV 13.3 ± 1.2 C 13.1 ± 1.3	IV 82 (38–106) C 81 (43–99)	IV 18.8 ± 6.0 C 19.4 ± 7.0	FCM	12
IRONMAN (Kalra el al.)	2022	569	568	IV 73.2 (66.7–80.1) C 73.5 (67.1–79.1)	IV 25 C 28	IV = 32% (IQR, 25–37) C = 35% (IQR, 26–38)	LVEF ≤ 45 NYHA Grade II/III/IV Hb 9–14	IV 52 C 55	IV 44 C 47	IV 50 C 44	IV 12.1 (11.2–12.8) C 12.1 (11.2–12.9)	IV 49.0 (30.0–86.0) C 50.0 (30.0–85.0)	IV 15 (11–20) C 15 (10–19)	FDI	140 (94–187)
FAIR‐HF (Anker et al.)	2009	304	155	IV 67.8 ± 10.3 C 67.4 ± 11.1	IV 52.3 C 54.8	IV = 31.9 ± 5.5 C = 33.0 ± 6.1	LVEF ≤ 45 NYHA Grade II/III Hb 9.5–13.5	IV 79.9 C 82.6	IV 30.6 C 23.9	IV 30.9 C 28.4	IV 119 ± 13 C 119 ± 14	IV 52.5 ± 54.5 C 60.1 ± 66.5	IV 17.7 ± 12.6 C 16.7 ± 8.4	FCM	24
CONFIRM‐HF (Ponikowski et al.)	2014	152	152	IV 68.8 ± 9.5 C 69.5 ± 9.3	IV 45 C 49	IV = 37.1 ± 7.5 C = 36.5 ± 7.3	LVEF ≤ 45 NYHA Grade II/III Hb < 15	IV 87 C 86	IV 25 C 305	IV 44 C 48	IV 12.37 ± 1.41 C 12.42 ± 1.30	IV 57.0 ± 48.4 C 57.1 ± 41.6	IV 20.2 ± 17.6 C 18.2 ± 8.1	FCM	52
AFFIRM‐AHF (Ponikowski et al.)	2020	558	550	IV 71.2 ± 10.8 C 70.9 ± 11.1	IV 44 C 45	IV = 32.6 ± 9.6 C = 32·7 ± 10·0	LVEF ≤ 50 NYHA Grade I/II/III/IV Hb 8–15	IV 84 C 86	IV 41 C 44	IV 56 C 55	IV 12.3 ± 1.6 C 12.1 ± 1.6	IV 83.9 ± 62.2 C 88.5 ± 68.6	IV 15.2 ± 8.3 C 14.2 ± 7.5	FCM	52

AF, atrial fibrillation; C, control (placebo/standard of care); CVD, cardiovascular death; DM, diabetes mellitus; F, ferritin; FCM, ferric carboxymaltose; FDI, ferric derisomaltose; Hb, haemoglobin; HHF, hospitalization for heart failure; HTN, hypertension; ID, iron deficiency; IIM, iron isomaltoside; IS, iron sucrose; IV, intravenous; LVEF, left ventricular ejection fraction; MI, myocardial infarction; NA, not available; NYHA, New York Heart Association; SFGC, sodium ferric gluconate complex; SOC, standard of care; TSAT, transferrin saturation.

### Quality assessment of included studies

Ten studies had an overall low risk of bias. The remaining four studies had some concerns of bias. The details of the bias assessment for each included study are shown in *Figure* *S1*.

### Results of the meta‐analysis

#### First heart failure hospitalization or cardiovascular death

**Figure 2:**
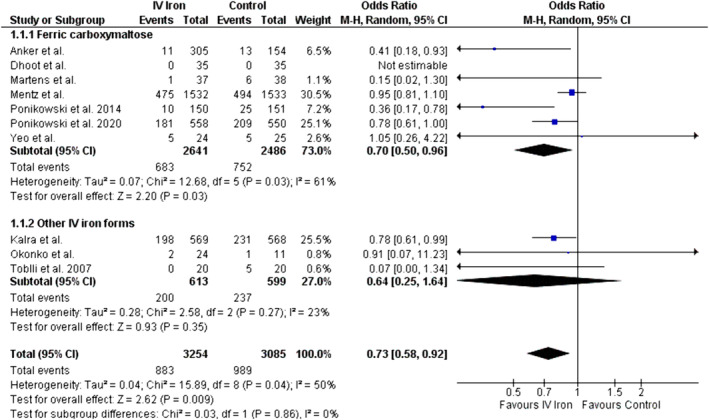
Forest plot for the composite outcome of the first HHF or CV death.

Data for the composite outcome of the first HHF or CV death were provided by 10 out of 14 included studies for the IV iron group (total, 3254; events, 883) and the control group (total, 3085; events, 989). The pooled analysis showed a significantly reduced incidence of first HHF or CV death in the IV iron group compared with the control group (OR = 0.73, 95% CI: 0.58 to 0.92, *P* = 0.009; *Figure* *2*). There was a moderate heterogeneity among the included studies (*I* ^2^ = 50%). The exclusive analysis of the seven studies in which ferric carboxymaltose was used and three studies in which other IV iron forms were used demonstrated a significant reduction in first HHF or CV death in the ferric carboxymaltose group (OR = 0.70, 95% CI = 0.50 to 0.96, *P* = 0.03; *I* ^2^ = 61%). However, with other IV iron forms, there was no significant difference between IV iron and the control group (OR = 0.64, 95% CI = 0.25 to 1.64, *P* = 0.35, *I* ^2^ = 23%). The difference between the two subgroups (ferric carboxymaltose vs. other IV iron forms) was insignificant (*P* = 0.86). Meta‐regression analysis showed that as baseline LVEF decreased, the effect of IV iron supplementation on first HHF or CV death became nonsignificant (*P =* 0.01; *Figure* *S2*). However, we observed no moderating effect of follow‐up duration (*P =* 0.45; *Figure* *S3*). Begg's test showed no significant risk of publication bias (*P* = 0.53).

#### Overall all‐cause mortality

**Figure 3:**
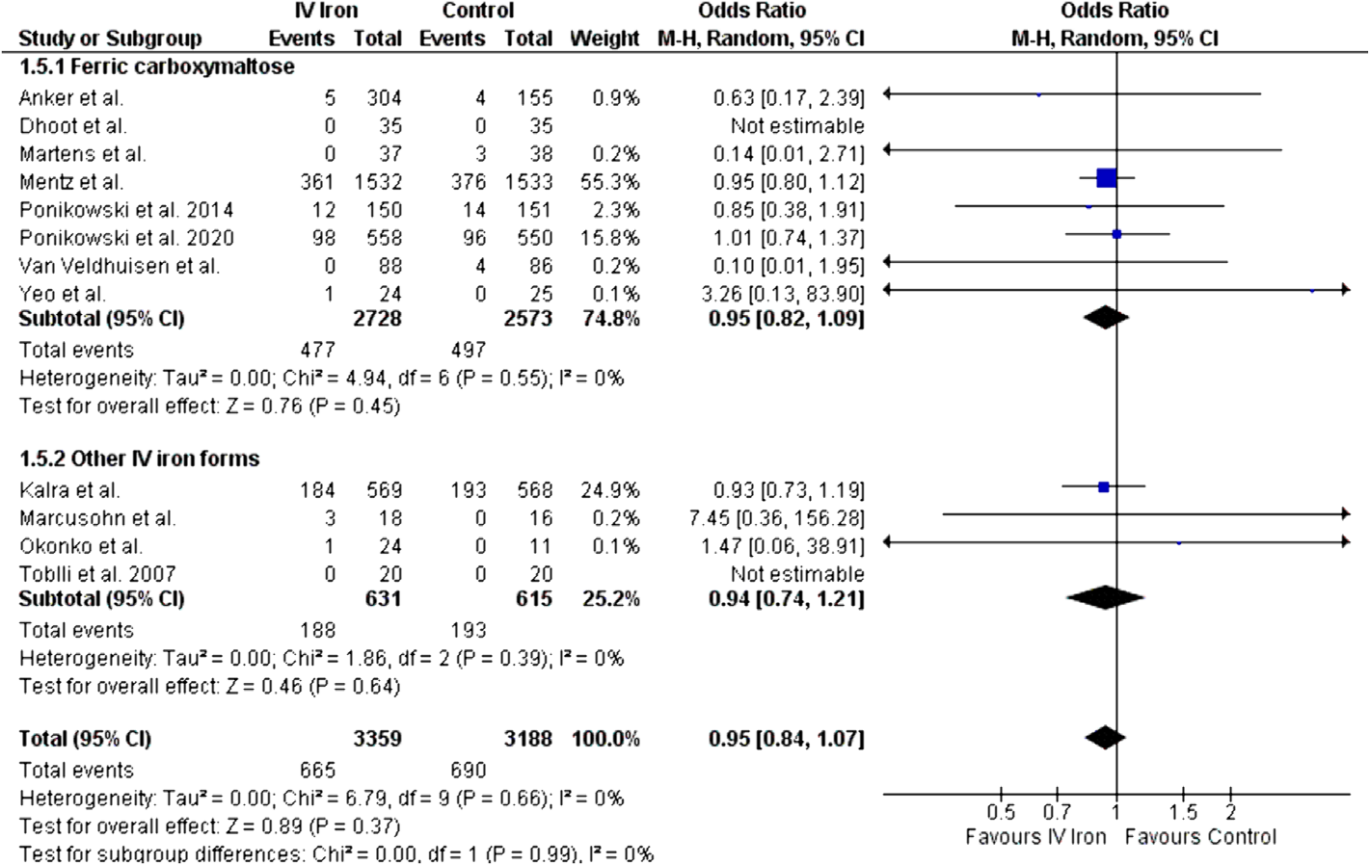
Forest plot for overall all‐cause mortality.

With data from 12 studies, our analysis demonstrated no significant difference in the incidence of all‐cause mortality between the treatment and control groups (OR = 0.95, 95% CI: 0.84 to 1.07, *P* = 0.37; *I* ^2^ = 0%; *Figure* *3*). Subgroup analysis showed no significant difference for reduction in all‐cause mortality with ferric carboxymaltose and other IV iron forms (ferric carboxymaltose: OR = 0.95, 95% CI = 0.82 to 1.09, *P* = 0.45, *I* ^2^ = 0%; other IV iron forms: OR = 0.94, 95% CI = 0.74 to 1.21, *P* = 0.64, *I* ^2^ = 0%,) The difference between the two subgroups (ferric carboxymaltose vs. other IV iron forms) was insignificant (*P* = 0.99). Meta‐regression showed no significant moderating effect of baseline LVEF (*P* = 0.87; *Figure* *S4*) and follow‐up (*P* = 0.91; *Figure* *S5*) on all‐cause mortality. Begg's rank test showed no significant risk of publication bias (*P* = 0.92).

#### Overall cardiovascular mortality

**Figure 4:**
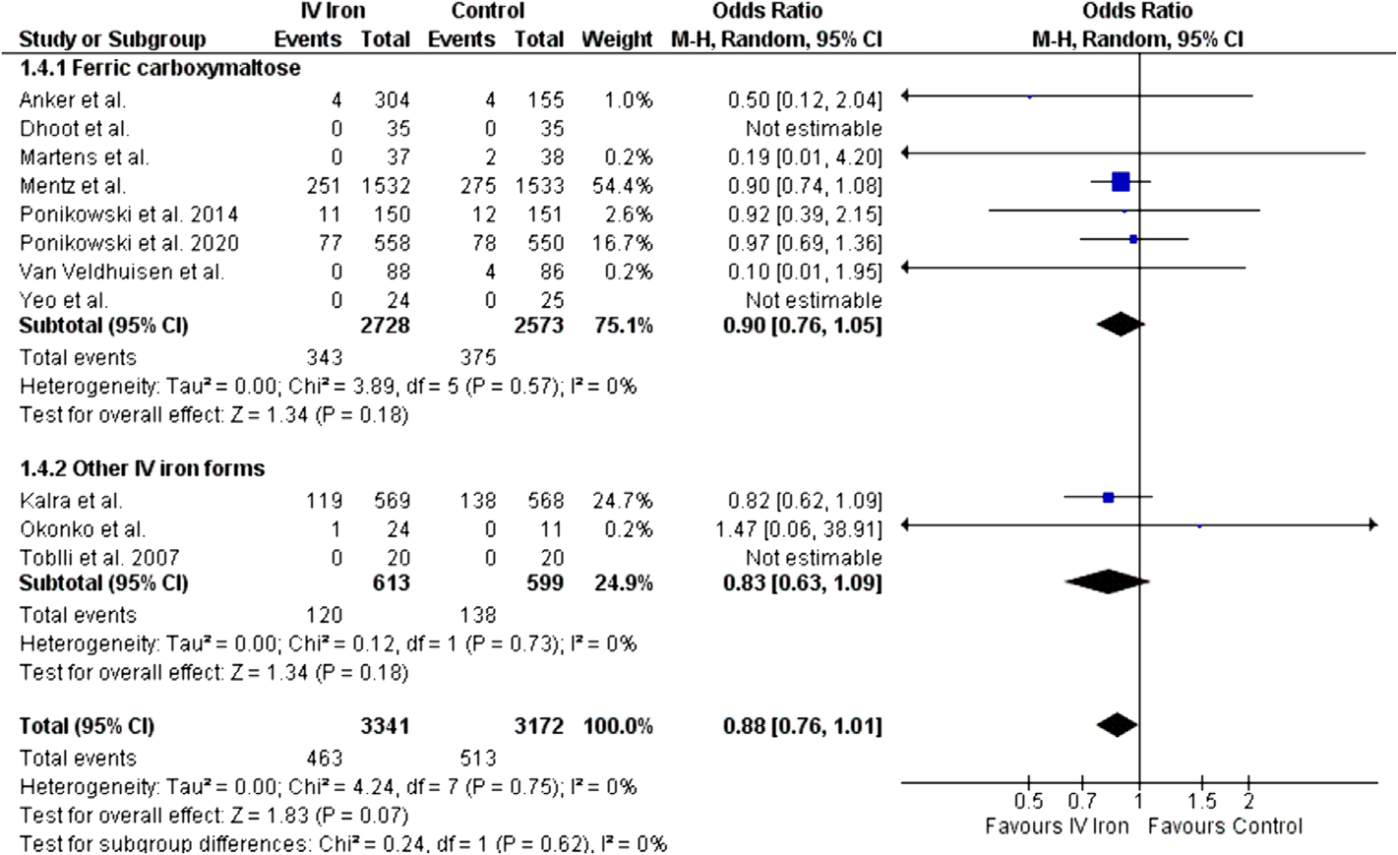
Forest plot for overall CV mortality.

Data from 11 studies showed a trend towards lower overall CV mortality in the IV iron group compared with the control group (OR = 0.88, 95% CI: 0.76 to 1.01, *P* = 0.07; *Figure* *4*). No heterogeneity was observed (*I* ^2^ = 0%). No statistically significant changes were observed on subgroup analysis (ferric carboxymaltose: OR = 0.90, 95% CI = 0.76 to 1.05, *P* = 0.18; *I* ^2^ = 0%; other IV iron forms: OR = 0.83, 95% CI = 0.63 to 1.09, *P* = 0.18; *I* ^2^ = 0%). The difference between the two subgroups was insignificant (*P* = 0.62). Meta‐regression showed no significant moderating effect of baseline LVEF (*P* = 0.93; *Figure* *S6*) and follow‐up (*P* = 0.70; *Figure* *S7*) on CV mortality. Begg's rank test showed no significant risk of publication bias (*P* = 0.21).

#### 1‐year all‐cause mortality

There was a trend towards lower 1‐year all‐cause mortality in the IV iron group (OR = 0.85, 95% CI 0.71 to 1.02, *P* = 0.07; *Figure* *S8*). Heterogeneity remained low (*I* ^2^ = 4%). The risk of publication bias was insignificant (*P* = 0.6).

#### 1‐year cardiovascular mortality

There was no significant difference between the 1‐year CV mortality in the IV iron and control groups (OR = 0.86, 95% CI: 0.71 to 1.04, *P* = 0.12; *Figure* *S9*). There was no observed heterogeneity (*I* ^2^ = 0%). Publication bias was insignificant (*P* = 0.6).

#### First heart failure hospitalization

Our pooled analysis showed a trend towards lower first HFF in the IV iron group (OR = 0.73, 95% CI: 0.51 to 1.05, *P* = 0.09; *Figure* *S10*). Moderate heterogeneity was observed for this outcome (*I* ^2^ = 60%). Publication bias was insignificant (*P* = 0.4).

#### Left ventricular ejection fraction

Our analysis showed a trend towards improvement in LVEF with IV iron (MD = 4.54, 95% CI: −0.13 to 9.21, *P* = 0.06; *Figure* *S11*). There was high heterogeneity (*I* ^2^ = 93%). Publication bias was insignificant (*P* = 0.6).

#### Improvement in New York Heart Association functional class

There was no significant difference in the improvement in NYHA class between the two groups (MD = −0.19, 95% CI: −1.62 to 1.23, *P* = 0.79; *Figure* *S12*). Heterogeneity was high for this outcome (*I* ^2^ = 98%). Publication bias was insignificant (*P* = 0.4).

#### 6‐min walk test

There was no significant improvement in the results of the 6‐min walk test among patients who received IV iron compared with the control group (MD = 19.87, 95% CI: −5.95 to 45.69, *P* = 0.13; *Figure* *S13*). A high heterogeneity was observed among the included studies (*I* ^2^ = 77%). Publication bias was insignificant (*P* = 0.1).

#### Adverse events

Five out of the 14 studies contributed to the analysis of adverse events, which included allergic reactions like rash, headache, nausea, abdominal pain, or any other neurological, gastrointestinal, or cardiovascular disorders. The pooled analysis revealed no significant differences between the IV iron and control groups in the incidence of adverse events (OR = 0.63, 95% CI: 0.36 to 1.09, *P* = 0.10; *Figure* *S14*). There was moderate heterogeneity (*I* ^2^ = 57%). Publication bias was insignificant (*P* = 1.0).

### Leave‐one‐out sensitivity analysis

We performed a leave‐one‐out sensitivity analysis for the outcomes that reported high heterogeneity (*I* ^2^ ≥ 75%). In the 6‐min walk test, excluding Kalra et al.^19^ reduced heterogeneity to *I* ^2^ = 72%, and the pooled estimates showed a statistically significant greater distance covered in the IV iron group as compared with the control group (MD = 30.25, 95% CI: 3.24 to 57.26, *P* = 0.03).

For LVEF, we performed sensitivity analysis by excluding Dhoot et al.,^25^ and heterogeneity reduced to *I* ^2^ = 0% with a significantly improved LVEF in the IV iron group (MD = 7.02, 95% CI: 5.58 to 8.47, *P* ≤ 0.00001). On performing sensitivity analysis for NYHA class improvement by excluding two studies,^19^, ^20^ heterogeneity was reduced to 96% and 89%, with no significant changes observed in overall pooled estimates. The details of sensitivity analysis are provided in *Table* *S2* and *Figures* *S15–S17*. The funnel plots for each pooled outcome are provided as *Figures* *S18–S27*.

## Discussion

This meta‐analysis of 14 RCTs encompassing 6651 patients sought to compare the safety and efficacy of IV iron administration in HF patients with concomitant iron deficiency. Our pooled analysis demonstrated a significantly reduced risk of the composite endpoint of first HHF and CV mortality compared with the control group. The pooled analysis showed a trend towards reduced CV mortality, 1‐year all‐cause mortality, and first HHF in the IV iron group. However, these endpoints did not reach statistical significance.

The results of this meta‐analysis are consistent with the results of a prior meta‐analysis^12^ that also concluded a significantly reduced risk of the composite endpoint of first HHF and CV mortality compared with the control group. However, our results showed a reduced trend for CV mortality, 1‐year‐all cause mortality, and first HHF with IV iron therapy without reaching statistical significance. There are a few possible explanations for these statistically nonsignificant results. Firstly, the inclusion of the recently published, largest clinical trial, the HEART‐FID trial,^14^ on the effects of IV iron substantially impacted the findings of our pooled analysis. The patient population in the HEART‐FID trial had a lower risk profile than the acute heart failure populations in the AFFIRM‐AHF trial,^11^ where every patient was enrolled while in the hospital, and the IRONMAN trial,^19^ where 15% of patients were included while in the hospital. Additionally, the HHF rate may have been impacted by the HEART‐FID trial's comparatively extensive use of evidence‐based drugs, particularly sacubitril‐valsartan.

Moreover, considering that most participants in the HEART‐FID study were enrolled during the COVID‐19 epidemic, the rate of hospitalizations and all‐cause mortality may have been impacted. This could be attributed to the COVID‐19 disruption, which made it challenging for many patients to receive repeated iron doses to sustain repletion. It is also plausible that non‐protocol IV iron delivery to control groups and oral iron supplements attenuated the impact of the intervention group observed in our included studies.

Considering the variability in the baseline LVEF of enrolled patients, we performed meta‐regression, which showed that a lower LVEF is associated with a decreased benefit of IV iron for the composite of first HHF or CV death. However, we observed no significant moderating effect of baseline LVEF on all‐cause and CV mortality. The follow‐up duration had no significant moderating effect on any primary outcome. Nevertheless, these findings may be considered hypothesis‐generating, and future randomized trials should investigate further to provide conclusive evidence.

The present meta‐analysis also evaluated symptomatic improvement defined by the evaluation of cardiac parameters, NYHA class and LVEF, and functional health improvement outcome measures of increase in exercise capacity defined as change in 6‐min walk test. The pooled estimates showed a trend towards improved LVEF, NYHA class, and 6‐min walk test without reaching statistical significance. The safety profile of IV iron was good, as evidenced by no increase in the incidence of adverse events compared with the placebo.

According to the 2021 ESC recommendations,^7^ all patients with HF should undergo routine full blood count, serum ferritin concentration, and transferrin saturation (TSAT) screening for anaemia and iron deficiency. Additionally, iron deficiency and/or anaemia should require a proper examination to determine their cause. The guidelines recommend that IV iron supplementation with ferric carboxymaltose should be taken into consideration in symptomatic patients with LVEF ≤45% and iron deficiency, defined as serum ferritin <100 ng/mL or serum ferritin 100–299 ng/mL with TSAT <20%, to relieve HF symptoms, improve exercise capacity, and enhance quality of life. Although the ESC recommendations currently only prescribe ferric carboxymaltose because of the abundance of evidence supporting this iron‐carbohydrate formulation, appropriately powered RCTs may support the use of alternative formulations for treating iron deficiency in patients with HF.

Although there are earlier meta‐analyses on this topic, our meta‐analysis offers the most recent and comprehensive overview as it includes all studies performed to date, including those that earlier meta‐analyses missed and those that have been published more recently. Additionally, we investigated the effect of potential effect modifiers like baseline LVEF and follow‐up duration, which prior reviews have not addressed. Thus, the current state of the literature on this topic and the total impact magnitude are accurately evaluated. Moreover, our pooled analysis includes 14 RCTs with a substantial cumulative sample size, providing greater statistical power and more reliable results. Additionally, we systematically evaluated the literature using well‐defined inclusion criteria, reasonable exclusion of duplicate research and studies with poorly interpreted results, and meticulous data extraction. We also performed a sensitivity analysis to address the high heterogeneity (>75%).

This study also has some limitations. Firstly, this is a study‐level meta‐analysis, and the absence of patient‐level information made it challenging to evaluate more effect modifiers. Secondly, significant heterogeneity was observed in some of the outcomes. This could be attributed to the variation in the length of the follow‐up period among the included studies. Moreover, the observed heterogeneity may have been influenced by variations in patient characteristics, such as some patients' concomitant kidney disease with HF.

## Conclusions

IV iron reduced the composite outcome of first HHF or CV death, specifically with the use of ferric carboxymaltose. Patients with lower baseline LVEF may have reduced benefit. IV iron therapy resulted in a trend towards lower overall CV and 1‐year all‐cause mortality, first HHF, and improved LVEF without reaching statistical significance. More evidence is needed to reach a definitive conclusion.

## Conflict of interest

Dr Fonarow reports consulting for Abbott, Amgen, AstraZeneca, Bayer, Cytokinetics, Edwards, Eli Lilly, Janssen, Medtronic, Merck, Novartis, and Pfizer. The rest of the authors report no relationships that could be construed as a conflict of interest.

## Supporting information


**Table S1.** List of common variable definitions.
**Table S2.** Results of leave‐one‐out sensitivity analysis of the variables with high heterogeneity (>75%).
**Figure S1.** Cochrane risk of bias (RoB 2.0) assessment for the included studies.
**Figure S2.** Meta‐regression plot showing the effect of baseline LVEF on the composite outcome of first hospitalization for HF (HHF) and cardiovascular (CV) death. Meta‐regression analysis showed that lower baseline LVEF is associated with a statistically significant increase in composite first HHF or CV death (*P =* 0.01). (Note: bubbles represent study, the size of bubble presents the weight of the study, and the central thick line presents meta‐regression line).
**Figure S3.** Meta‐regression plot showing the effect of follow‐up on the composite outcome of first hospitalization for HF (HHF) and cardiovascular (CV) death. Meta‐regression showed a statistically nonsignificant moderating effect of follow‐up (*P* = 0.45). (Note: bubbles represent study, the size of bubble presents the weight of the study, and the central thick line presents meta‐regression line).
**Figure S4.** Meta‐regression plot showing the effect of baseline LVEF on the overall all‐cause mortality. Meta‐regression showed a statistically nonsignificant moderating effect of baseline LVEF on all‐cause mortality (*P* = 0.87). (Note: bubbles represent study, the size of bubble presents the weight of the study, and the central thick line presents meta‐regression line).
**Figure S5.** Meta‐regression plot showing the effect of follow‐up on overall all‐cause mortality. Meta‐regression showed a statistically nonsignificant moderating effect of follow‐up (*P* = 0.91). (Note: bubbles represent study, the size of bubble presents the weight of the study, and the central thick line presents meta‐regression line).
**Figure S6.** Meta‐regression plot showing the effect of baseline LVEF on the overall CV mortality. Meta‐regression showed a statistically nonsignificant moderating effect of baseline LVEF on CV mortality (*P* = 0.93). (Note: bubbles represent study, the size of bubble presents the weight of the study, and the central thick line presents meta‐regression line).
**Figure S7.** Meta‐regression plot showing the effect of follow‐up on overall all‐cause mortality. Meta‐regression showed a statistically nonsignificant moderating effect of follow‐up (*P* = 0.70). (Note: bubbles represent study, the size of bubble presents the weight of the study, and the central thick line presents meta‐regression line).
**Figure S8.** Forest plot for 1‐year all‐cause mortality with IV iron therapy.
**Figure S9.** Forest plot for 1‐year Cardiovascular (CV) mortality with IV iron therapy.
**Figure S10.** Forest plot for first Heart Failure Hospitalization (first HHF) with IV iron therapy.
**Figure S11.** Forest plot for improvement in left ventricular ejection fraction (LVEF) with IV iron therapy.
**Figure S12.** Forest plot for Improvement in NYHA functional class with IV iron therapy.
**Figure S13.** Forest plot for improvement in 6‐minute walk test with IV iron therapy.
**Figure S14.** Forest plot for adverse events with IV iron therapy.
**Figure S15.** Results of leave‐one‐out analysis for 6‐min walk distance.
**Figure S16.** Results of leave‐one‐out analysis for Left Ventricular Ejection.
**Figure S17.** Results of leave‐one‐out analysis for New York Heart Association Class Improvement.
**Figure S18.** Funnel plot assessing the risk of publication bias for the composite outcome of first heart failure hospitalization and cardiovascular death.
**Figure S19.** Funnel plot assessing the risk of publication bias for overall all‐cause mortality.
**Figure S20.** Funnel plot assessing the risk of publication bias for overall cardiovascular (CV) mortality.
**Figure S21.** Funnel plot assessing the risk of publication bias for 6‐minute walk test.
**Figure S22.** Funnel plot assessing the risk of publication bias for 1‐year CV mortality.
**Figure S23.** Funnel plot assessing the risk of publication bias for 1‐year all‐cause mortality.
**Figure S24.** Funnel plot assessing the risk of publication bias for first HF hospitalization.
**Figure S25.** Funnel plot assessing the risk of publication bias for LVEF.
**Figure S26.** Funnel plot assessing the risk of publication bias for NYHA class improvement.
**Figure S27.** Funnel plot assessing the risk of publication bias for Adverse events.

## Data Availability

The data supporting this study's findings are available from the corresponding author upon reasonable request.
