# Supplementary material for: Intravenous iron therapy for heart failure and iron deficiency: An updated meta‐analysis of randomized clinical trials
Source: ESC Heart Fail. 2024 Jul 4;12(1):43–53. doi: 10.1002/ehf2.14905 (PMC11769671; doi:10.1002/ehf2.14905)
Supplement: Supplementary file 1 — Table S1. List of common variable definitions. Table S2. Results of leave‐one‐out sensitivity analysis of the variables with high heterogeneity (>75%). Figure S1. Cochrane risk of bias (RoB 2.0) assessment for the included studies. Figure S2. Meta‐regression plot showing the effect of baseline LVEF on the composite outcome of first hospitalization for HF (HHF) and cardiovascular (CV) death. Meta‐regression analysis showed that lower baseline LVEF is associated with a statistically significant increase in composite first HHF or CV death (P = 0.01). (Note: bubbles represent study, the size of bubble presents the weight of the study, and the central thick line presents meta‐regression line). Figure S3. Meta‐regression plot showing the effect of follow‐up on the composite outcome of first hospitalization for HF (HHF) and cardiovascular (CV) death. Meta‐regression showed a statistically nonsignificant moderating effect of follow‐up (P = 0.45). (Note: bubbles represent study, the size of bubble presents the weight of the study, and the central thick line presents meta‐regression line). Figure S4. Meta‐regression plot showing the effect of baseline LVEF on the overall all‐cause mortality. Meta‐regression showed a statistically nonsignificant moderating effect of baseline LVEF on all‐cause mortality (P = 0.87). (Note: bubbles represent study, the size of bubble presents the weight of the study, and the central thick line presents meta‐regression line). Figure S5. Meta‐regression plot showing the effect of follow‐up on overall all‐cause mortality. Meta‐regression showed a statistically nonsignificant moderating effect of follow‐up (P = 0.91). (Note: bubbles represent study, the size of bubble presents the weight of the study, and the central thick line presents meta‐regression line). Figure S6. Meta‐regression plot showing the effect of baseline LVEF on the overall CV mortality. Meta‐regression showed a statistically nonsignificant moderating effect of baseline LVEF on CV mo [file EHF2-12-43-s001.docx]

Supplementary Appendix

Supplementary Table 1: List of Common Variable Definitions

Supplementary Table 2: Results of leave-one-out sensitivity analysis of the variables with high heterogeneity (>75%).

Supplementary Figure 1: Cochrane risk of bias (RoB 2.0) assessment for the included studies

Supplementary Figure 2: Meta-regression plot showing the effect of baseline left ventricular ejection fraction (LVEF) on the composite outcome of first hospitalization for HF (HHF) and cardiovascular (CV) death

Supplementary Figure 3: Meta-regression plot showing the effect of follow-up (weeks) on the composite outcome of first hospitalization for HF (HHF) and cardiovascular (CV) death

Supplementary Figure 4: Meta-regression plot showing the effect of baseline left ventricular ejection fraction (LVEF) on overall all-cause mortality

Supplementary Figure 5: Meta-regression plot showing the effect of follow-up (weeks) on overall all-cause mortality

Supplementary Figure 6: Meta-regression plot showing the effect of baseline left ventricular ejection fraction (LVEF) on overall CV mortality

Supplementary Figure 7: Meta-regression plot showing the effect of follow-up (weeks) on overall CV mortality

Supplementary Figure 8: Forest plot for 1-year all-cause mortality with IV iron therapy

Supplementary Figure 9: Forest plot for 1-year Cardiovascular (CV) mortality with IV iron therapy

Supplementary Figure 10: Forest plot for first Heart Failure Hospitalization (first HHF) with IV iron therapy

Supplementary Figure 11: Forest plot for improvement in left ventricular ejection fraction (LVEF) with IV iron therapy

Supplementary Figure 12: Forest plot for Improvement in NYHA functional class with IV iron therapy

Supplementary Figure 13: Forest plot for improvement in 6-minute walk test with IV iron therapy

Supplementary Figure 14: Forest plot for adverse events with IV iron therapy

Supplementary Figure 15: Results of leave-one-out analysis for 6-min walk distance

Supplementary Figure 16: Results of leave-one-out analysis for Left Ventricular Ejection Fraction

Supplementary Figure 17: Results of leave-one-out analysis for New York Heart Association Class Improvement

Supplementary Figures 18-27: Funnel plot assessing the risk of publication bias each outcome

Supplementary Table 1: List of common variable definitions

| Common Variables | Definitions. |
| --- | --- |
| Heart failure | A chronic medical condition in which the heart is unable to pump blood efficiently, leading to inadequate oxygen supply to the body. |
| First heart failure hospitalization | The first occurrence of a patient with heart failure being admitted to a hospital due to worsening symptoms and complications. |
| Cardiovascular mortality | The rate of death from heart-related causes, including heart attacks, heart failure, and other cardiovascular diseases. |
| All-cause mortality | The rate of death from all possible causes, including cardiovascular diseases, cancer, infections, accidents, and others. |
| Left ventricular ejection fraction | A measurement of the percentage of blood ejected from the left ventricle of the heart during each contraction (systole). |
| 6-minute walk test | A test that measures a patient's exercise tolerance and functional capacity by assessing how far they can walk in 6 minutes. |
| Iron deficiency | A condition characterized by a lack of sufficient iron in the body, which can lead to anemia and other health problems |
| Adverse Events (AEs): | Undesirable and unintended outcomes or side effects resulting from medical treatments or interventions. |

Supplementary Table 2: Results of leave-one-out sensitivity analysis of the variables with high heterogeneity (>75%).

| Study removed | Heterogeneity with study removed | MD (95% CI) | *P* value |
| --- | --- | --- | --- |
| **A. 6 minute-walk test** |  |  |  |
| Dhoot et al. | I^2^ = 72%, *P* = 0.006 | 13.60 (-14.17 to 41.36) | 0.34 |
| Kalra et al. | I^2^ = 75%, *P* = 0.003 | 30.25 (3.24 to 57.26) | 0.03 |
| Mentz et al. | I^2^ = 73%, *P* = 0.005 | 24.84 (-9.21 to 57.26) | 0.15 |
| Toblli et al. 2007 | I^2^ = 71%, *P* = 0.008 | 12.27 (-13.57 to 38.11) | 0.35 |
| **B. Left Ventricular Ejection Fraction** |  |  |  |
| Dhoot et al. | I^2^ = 0%, *P* = 0.89 | 7.02 (5.58 to 8.47) | <0.00001 |
| **C. Improvement in New York Heart Association (NYHA) class** |  |  |  |
| Toblli et al. 2017 | I^2^ = 96%, *P* = <0.00001 | 0.19 (-1.06 to 1.44) | 0.76 |
| Toblli et al. 2015 | I^2^ = 89%, *P* = 0.00001 | -0.72 (-1.50 to 0.06) | 0.07 |

Supplementary Figure 1: Cochrane risk of bias (RoB 2.0) assessment for the included studies


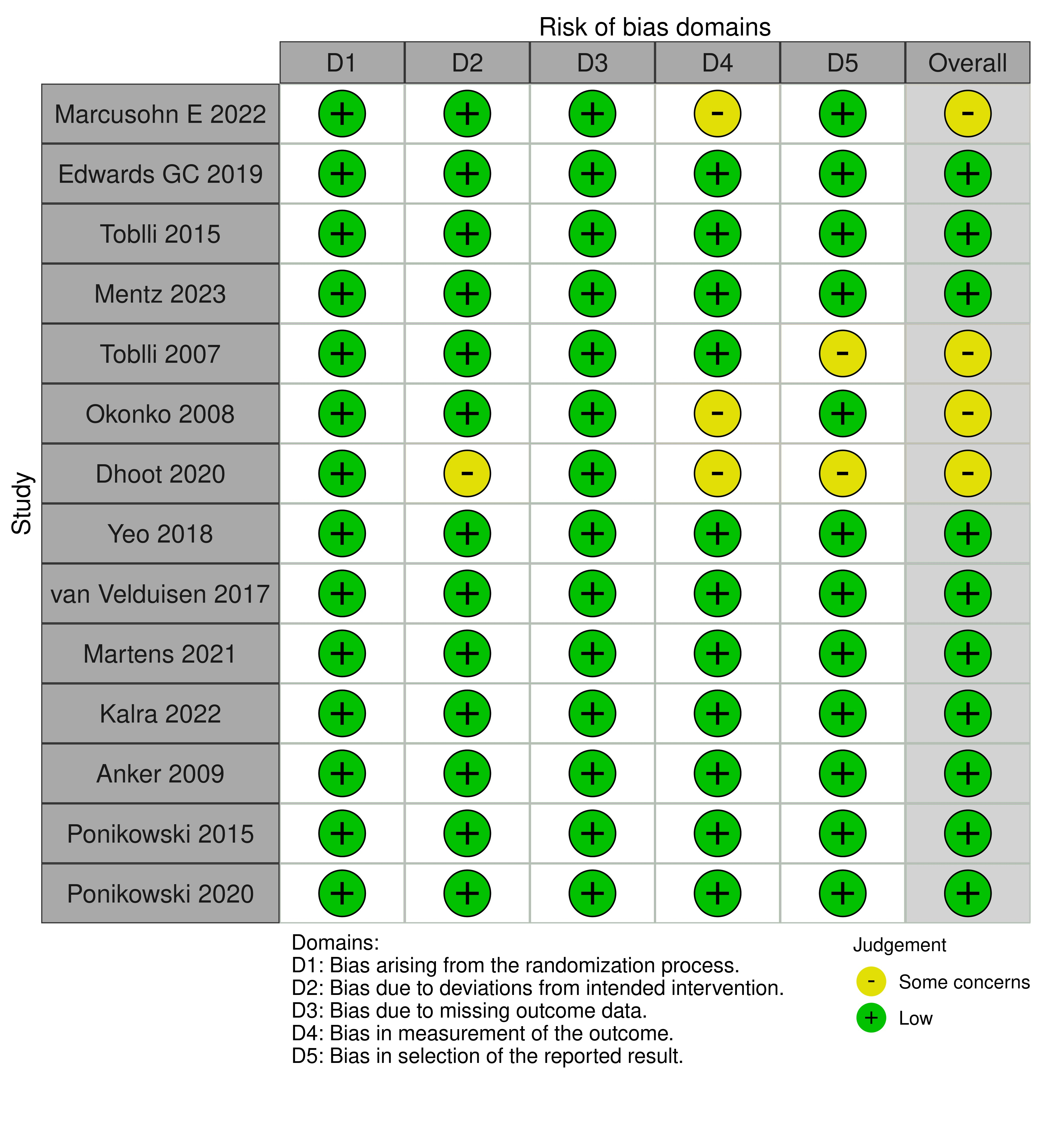


Supplementary Figure 2: Meta-regression plot showing the effect of baseline LVEF on the composite outcome of first hospitalization for HF (HHF) and cardiovascular (CV) death. Meta-regression analysis showed that lower baseline LVEF is associated with a statistically significant increase in composite first HHF or CV death (*p =* 0.01). (Note: bubbles represent study, the size of bubble presents the weight of the study, and the central thick line presents meta-regression line).

Supplementary Figure 3: Meta-regression plot showing the effect of follow-up on the composite outcome of first hospitalization for HF (HHF) and cardiovascular (CV) death. Meta-regression showed a statistically nonsignificant moderating effect of follow-up (*p* = 0.45). (Note: bubbles represent study, the size of bubble presents the weight of the study, and the central thick line presents meta-regression line).

Supplementary Figure 4: Meta-regression plot showing the effect of baseline LVEF on the overall all-cause mortality. Meta-regression showed a statistically nonsignificant moderating effect of baseline LVEF on all-cause mortality (*p* = 0.87). (Note: bubbles represent study, the size of bubble presents the weight of the study, and the central thick line presents meta-regression line).

Supplementary Figure 5: Meta-regression plot showing the effect of follow-up on overall all-cause mortality. Meta-regression showed a statistically nonsignificant moderating effect of follow-up (*p* = 0.91). (Note: bubbles represent study, the size of bubble presents the weight of the study, and the central thick line presents meta-regression line).

Supplementary Figure 6: Meta-regression plot showing the effect of baseline LVEF on the overall CV mortality. Meta-regression showed a statistically nonsignificant moderating effect of baseline LVEF on CV mortality (*p* = 0.93). (Note: bubbles represent study, the size of bubble presents the weight of the study, and the central thick line presents meta-regression line).

Supplementary Figure 7: Meta-regression plot showing the effect of follow-up on overall all-cause mortality. Meta-regression showed a statistically nonsignificant moderating effect of follow-up (*p* = 0.70). (Note: bubbles represent study, the size of bubble presents the weight of the study, and the central thick line presents meta-regression line).

Supplementary Figure 8: Forest plot for 1-year all-cause mortality with IV iron therapy


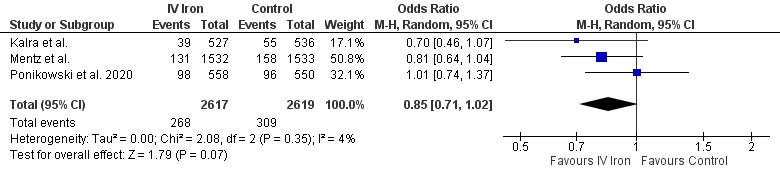


Supplementary Figure 9: Forest plot for 1-year Cardiovascular (CV) mortality with IV iron therapy


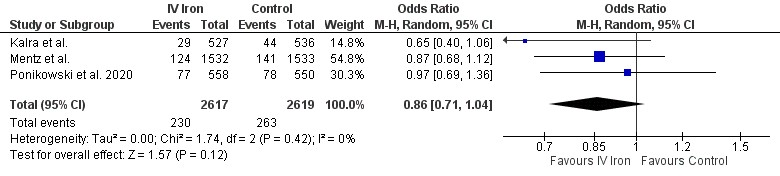


Supplementary Figure 10: Forest plot for first Heart Failure Hospitalization (first HHF) with IV iron therapy


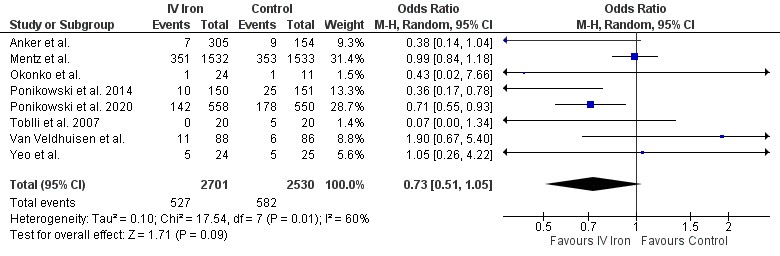


Supplementary Figure 11: Forest plot for improvement in left ventricular ejection fraction (LVEF) with IV iron therapy


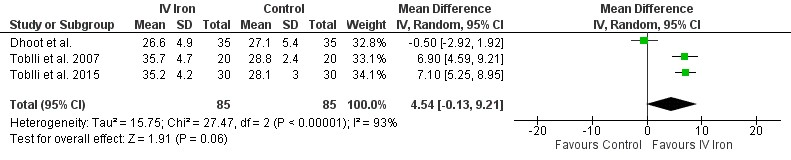


Supplementary Figure 12: Forest plot for Improvement in NYHA functional class with IV iron therapy


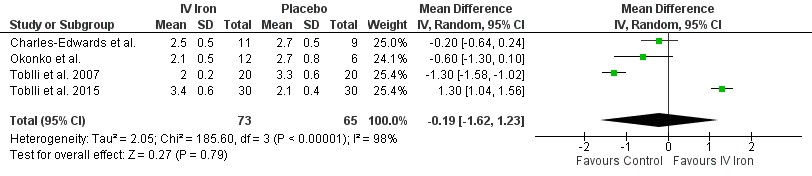


Supplementary Figure 13: Forest plot for improvement in 6-minute walk test with IV iron therapy


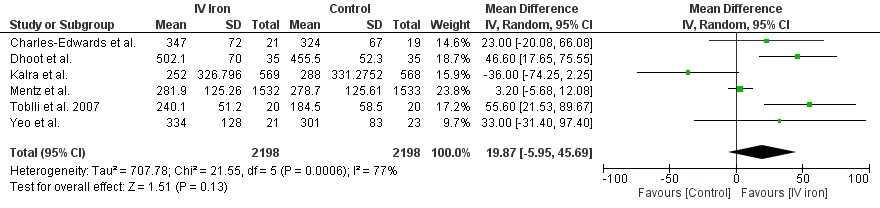


Supplementary Figure 14: Forest plot for adverse events with IV iron therapy


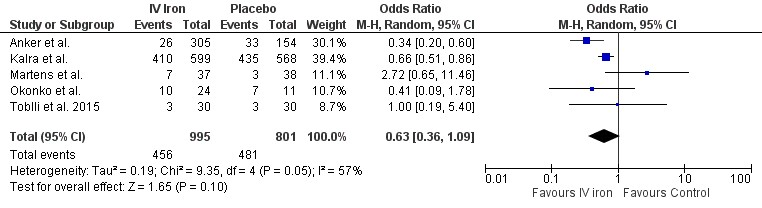


Supplementary Figure 15: Results of leave-one-out analysis for 6-min walk distance

1) On excluding Dhoot et al.


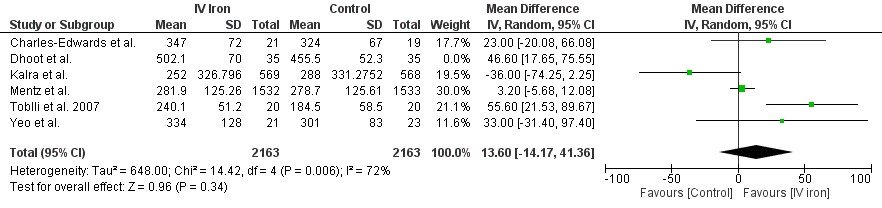
2) On excluding Kalra et al.
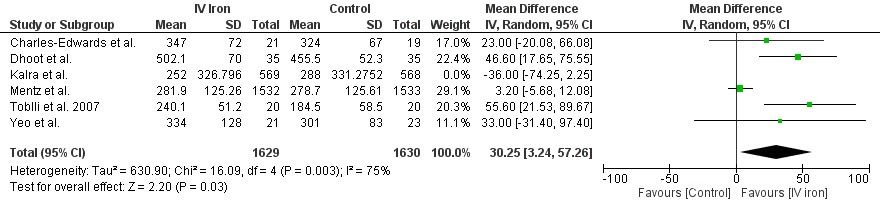


3) On excluding Mentz et al.


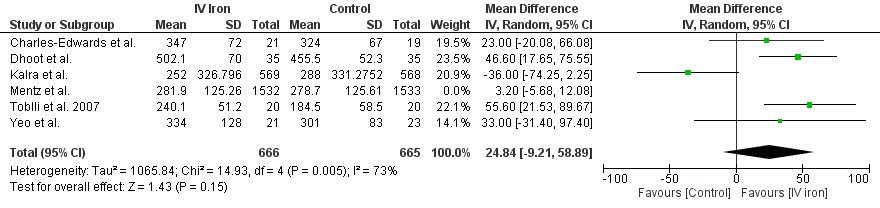


4) On excluding Toblli et al. 2007


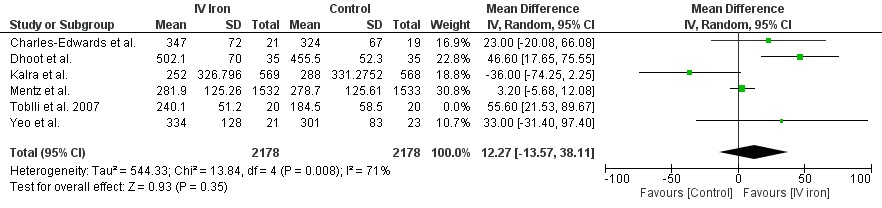


Supplementary Figure 16: Results of leave-one-out analysis for Left Ventricular Ejection Fraction


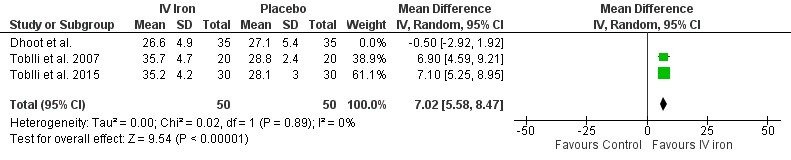


Supplementary Figure 17: Results of leave-one-out analysis for New York Heart Association Class Improvement

1) On excluding Toblli et al. 2007


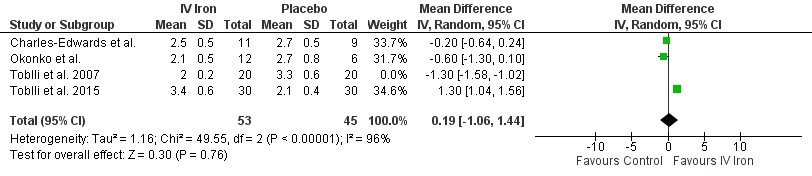


2) On excluding Toblli et al. 2015


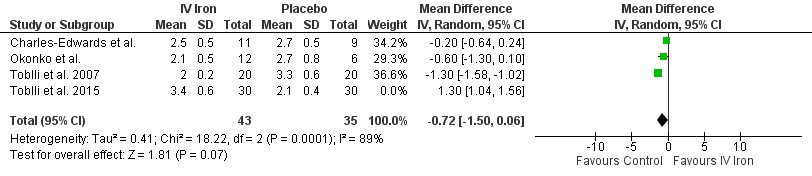


Supplementary Figure 18: Funnel plot assessing the risk of publication bias for the composite outcome of first heart failure hospitalization and cardiovascular death

Supplementary Figure 19: Funnel plot assessing the risk of publication bias for overall all-cause mortality

Supplementary Figure 20: Funnel plot assessing the risk of publication bias for overall cardiovascular (CV) mortality

Supplementary Figure 21: Funnel plot assessing the risk of publication bias for 6-minute walk test

Supplementary Figure 22: Funnel plot assessing the risk of publication bias for 1-year CV mortality

Supplementary Figure 23: Funnel plot assessing the risk of publication bias for 1-year all-cause mortality

Supplementary Figure 24: Funnel plot assessing the risk of publication bias for first HF hospitalization

Supplementary Figure 25: Funnel plot assessing the risk of publication bias for LVEF

Figure 26: Funnel plot assessing the risk of publication bias for NYHA class improvement

Supplementary Figure 27: Funnel plot assessing the risk of publication bias for Adverse events
